# Supplementary material for: Venomix: a simple bioinformatic pipeline for identifying and characterizing toxin gene candidates from transcriptomic data
Source: PeerJ. 2018 Jul 31;6:e5361. doi: 10.7717/peerj.5361 (PMC6074769; doi:10.7717/peerj.5361)
Supplement: Supplemental Information 3 [file peerj-06-5361-s003.gz › FinalOutput_E-20/Acetylcholinesterase-1_1/finaltree.pdf]

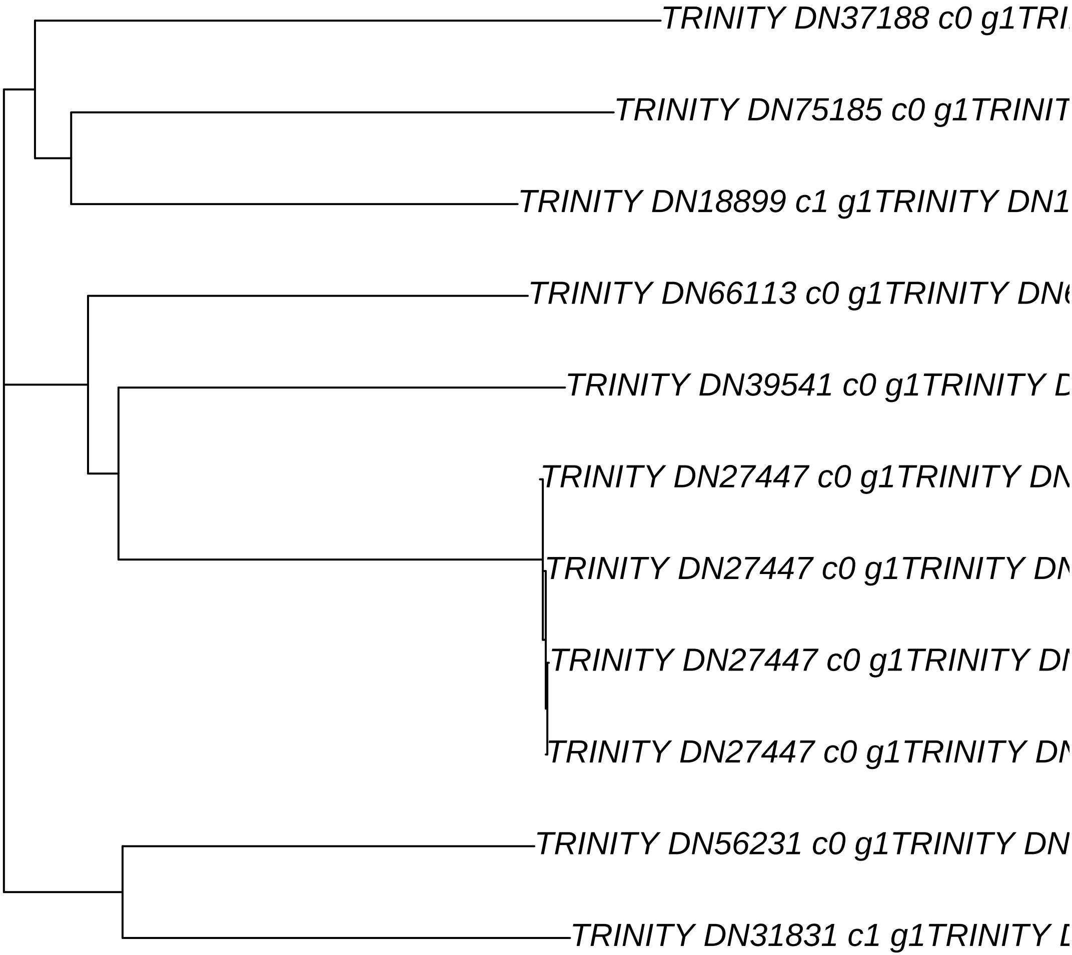

TRINITY DN37188 c0 g1TRI
TRINITY DN75185 c0 g1TRINIT
TRINITY DN18899 c1 g1TRINITY DN1
TRINITY DN66113 c0 g1TRINITY DN
TRINITY DN39541 c0 g1TRINITY D
TRINITY DN27447 c0 g1TRINITY DN
TRINITY DN27447 c0 g1TRINITY DN
TRINITY DN27447 c0 g1TRINITY DI
TRINITY DN27447 c0 g1TRINITY DN
TRINITY DN56231 c0 g1TRINITY DN
TRINITY DN31831 c1 g1TRINITY D
